# Morphofunctional cardiac changes in singleton and twin pregnancies: a longitudinal cohort study

**DOI:** 10.1186/s12884-020-03452-3

**Published:** 2020-12-02

**Authors:** Takeshi Umazume, Takahiro Yamada, Itsuko Furuta, Hiroyuki Iwano, Mamoru Morikawa, Hidemichi Watari, Hisanori Minakami

**Affiliations:** 1 Department of Obstetrics and Gynecology, Hokkaido University Graduate School of Medicine, N15W7, Kita-ku, Sapporo, 060-8638 Japan; 2 Department of Cardiovascular Medicine, Hokkaido University Graduate School of Medicine, Sapporo, Japan

**Keywords:** Echocardiography, Troponin, Brain natriuretic peptide, Diastolic function

## Abstract

**Background:**

This study aimed to compare the echocardiographic changes and cardiac biomarkers between women with singleton and twin pregnancies.

**Methods:**

From April 2014 to March 2016, this longitudinal cohort study invited pregnant women who were scheduled to give birth at Hokkaido University Hospital. We analyzed prospectively collected data on simultaneously determined echocardiographic parameters and blood cardiac markers of 44 women with singleton and 22 women with twin pregnancies. Furthermore, we tested the mixed-effect models for echocardiographic parameters and cardiac biomarkers.

**Results:**

During the third trimester and immediately postpartum (within 1 week after childbirth), the mean left atrial volume index and brain natriuretic peptide (BNP) level were significantly higher in women with twin pregnancies than in those with singleton pregnancies. Women with twin pregnancies also had significantly smaller second-trimester inferior vena cava diameters and significantly higher third^−^trimester creatinine levels than those with singleton pregnancies. BNP positively correlated with the left atrial volume index (β = 0.49, *p* < 0.01) and the ratio of early diastolic transmitral to mitral annular velocity (E/e’) (β = 0.41, *p* < 0.01). At 1 month after childbirth in women with singleton pregnancies, BNP and N-terminal precursor protein BNP (NT-proBNP) fragments immediately postpartum negatively correlated with the later E/e’ (r = − 0.33, *p* = 0.02 and r = − 0.36, *p* < 0.01, respectively).

**Conclusions:**

The intravascular cardiac load reached maximum within 1 week after childbirth and was greater in women with twin pregnancies than in those with singleton pregnancies. BNP/NT-proBNP significantly positively correlated with LA volume index and E/e’. In women with singleton pregnancies, BNP secreted immediately after childbirth might improve the diastolic functions 1 month after childbirth.

## Background

Twin pregnancy differs from singleton pregnancy in many aspects. Women with twin pregnancies have greater physiological increase in circulating blood volume [1], greater gestational weight gain and newborn weight (sum for twins) [2], shorter pregnancy length [3], more persistent systolic dysfunction after childbirth [4], and greater risk for peripartum cardiomyopathy than those with singleton pregnancies [5, 6].

In pregnancy, blood volume increases physiologically [1]; this volume expansion is associated with cardiac morphofunctional changes during gestation [7–12]. In Pritchard’s classical study [1], the circulating blood volume in singleton and twin pregnancies increased by 1570 and 1960 mL, respectively. As a result, the echocardiographic findings [13–17] and blood levels of cardiac biomarkers, such as B-type natriuretic peptide (BNP), N-terminal precursor protein BNP fragment (NT-proBNP), and high-sensitivity troponin I (hs-TnI), are significantly different between women with singleton and twin pregnancies [18, 19]. Kuleva et al. [16] revealed a higher cardiac output in twin pregnancies than in singleton pregnancies. According to Ghi et al., maternal systolic and diastolic functions change in all trimesters, and the systolic function decreases persistently after childbirth [17]. The BNP levels increase as heart failure worsens; thus, BNP level measurement is widely used to assess the presence, severity, and prognosis of heart failure [20, 21]. Both BNP and atrial natriuretic peptide are beneficial in patients with heart failure [22, 23]. However, the echocardiographic parameters and cardiac biomarker levels in singleton and twin pregnancies have not yet been compared, and their associations are still unknown.

## Methods

### Aim, design, and setting of the study

This study aimed to characterize the cardiac morphofunctional changes in normotensive women with twin or singleton pregnancies and to determine their associations with BNP, NT-proBNP, and hs-TnI levels using longitudinal prospectively collected data.

**Fig. 1:**
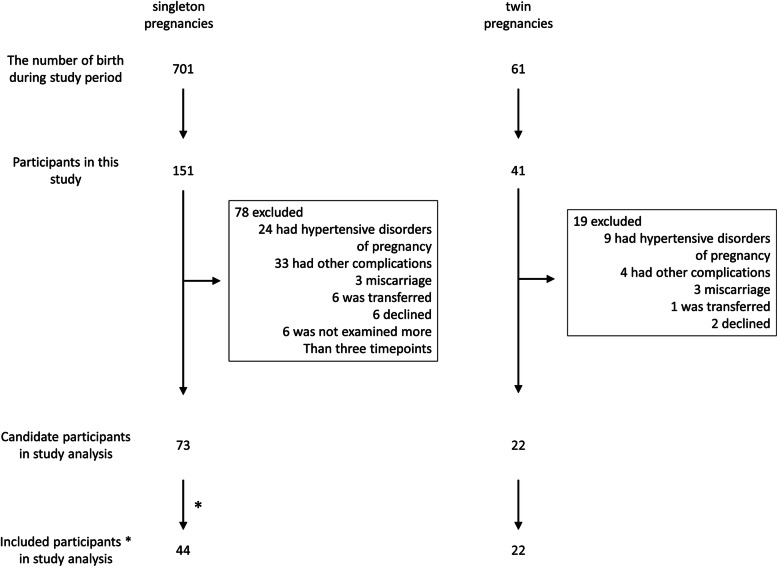
Study design. *, We selected two women of similar age with singleton pregnancies whose delivery date was closest to that of each woman with twin pregnancy who met the four criteria (see method) to serve as controls

This was a longitudinal cohort study. From April 2014 to March 2016, this study invited pregnant women who were scheduled to give birth at Hokkaido University Hospital. All participants were requested to undergo echocardiography and blood sampling in each trimester, within 1 week after childbirth (designated as “PP1”), and 1 month after childbirth (postpartum days 23–39) (designated as “PP2”). Out of 701 women with singleton pregnancies and 61 with twin pregnancies who gave birth at the hospital during the two-year study period, 151 and 41, respectively, participated in simultaneous echocardiography and blood samplings (Fig. 1). This study was conducted as an additional examination of our previous studies [11, 24].

### Participants (Fig. 1) [11, 24]

To compare longitudinal changes in echocardiographic findings between normotensive women with singleton and twin pregnancies, we selected 22 eligible participants with twin pregnancies who met all of the following four criteria: (1) no preexisting hypertension or development of hypertension during the current pregnancy; (2) no preexisting diseases, including hematologic, endocrine, and connective tissue diseases; (3) uneventful clinical course during the current pregnancy; and (4) simultaneous echocardiography and blood sampling at least thrice during the four stages of pregnancy/postpartum including the second trimester, third trimester, PP1, and PP2 time points. For the controls, we selected two women of similar age from three women with singleton pregnancies whose delivery date was closest to that of each eligible woman with twin pregnancy. A total of 44 women with singleton pregnancies were included.

### Echocardiographic evaluations [11, 24]

Each participant was placed in the left lateral decubitus position and underwent bedside transthoracic echocardiography by a single trained operator (TU) using the same machine (ProSound α7; Hitachi, Tokyo, Japan) according to the European Association of Cardiovascular Imaging guidelines [25]. We then calculated the stroke volume by multiplying the aortic valve area by the aortic flow velocity time integral. Only the inferior vena cava (IVC) diameter was evaluated in the dorsal position. To avoid any possible performance bias, the trained operator was blinded to the biochemical data while performing echocardiography.

### Biochemical procedures [11, 24]

We stored serum and plasma samples at − 20 °C before performing the assays of hs-TnI, NT-proBNP, BNP, and creatinine. To obtain the estimated glomerular filtration rate (eGFR), we used the following equation: 0.739 × 194 × serum creatinine^− 1.094^ × age [years]^− 0.287^ [26]. Furthermore, we measured the hs-TnI, BNP, and NT-proBNP levels using counting immunoassay kits (ARCHITECT High-Sensitivity Troponin I™, ARCHITECT BNP-JP™; Abbott Japan, Chiba, Japan; and Elecsys proBNP II STAT Assay™; Roche Diagnostics K.K., Tokyo, Japan, respectively).

### Statistical analysis [11, 24]

All statistical data were analyzed using JMP Pro12© (SAS, Cary, NC, USA) and SPSS version 24.0 (IBM, Armonk, NY, USA). Using Student’s *t*-test with Bonferroni correction, we compared changes in variables within a group. Correlations at each time point and associations between echocardiographic parameters and biomarker levels were assessed using the mixed-effects model. In addition, we standardized dependent (echocardiographic parameters) and independent (biomarker levels) variables to interpret the regression coefficient as the correlation coefficient. A compound symmetry covariance structure was implemented at each time point. Furthermore, *p* < 0.05 was considered to be statistically significant. However, in this study, a linear correlation between two standardized variables was considered significant if *p* was < 0.05 and standardized regression coefficient (β) was > 0.3 or ≤ 0.3.

## Results

Overall, 44 women with singleton pregnancies and 22 with twin pregnancies underwent 166 and 83 echocardiographic procedures along with blood sampling, respectively (Table 1). Women with twin pregnancies had a significantly higher percentage of gestational weight gains during third trimester than those with singleton pregnancies (Table 2).

**Table 1:** Demographic characteristics of the study population

	Singleton pregnancy	Twin pregnancy	*P-*value
No. of women	44	22	
Nulliparous women	21 (48%)	14 (64%)	0.222
Age (year)	32.2 ± 4.5	31.5 ± 5.5	0.754
Pre-pregnancy BMI (kg/m^2^)	21.5 ± 3.4	20.1 ± 2.2	0.128
Pre-pregnancy BSA (m^2^)	1.54 ± 0.10	1.48 ± 0.11	0.023
Gestational week at delivery	38.8 ± 1.4	36.7 ± 1.4	< 0.001
Cesarean delivery	18 (41%)	21 (95%)	< 0.001
Chorionicity			
monochorionic diamniotic		12	
dichorionic diamniotic		10	
Newborn weight, kg^a^	3.1 ± 0.4	4.4 ± 0.6	< 0.001
No. of tests^b^ given in 66 women	166	83	
No. of the tests^b^ per woman	3.8 ± 0.4	3.8 ± 0.4	1.000
Timing of the tests^b^			
Second trimester, weeks	25.3 ± 1.0 [42]	25.0 ± 1.0 [18]	0.202
Third trimester, weeks	35.7 ± 2.8 [42]	35.4 ± 0.4 [21]	0.709
PP1 (postpartum day 2–7)	3.2 ± 0.8 [41]	3.6 ± 1.1 [22]	0.171
PP2 (postpartum day 23–39)	31.0 ± 3.9 [41]	30.8 ± 2.7 [22]	0.901

Data are presented as the means ± SD. Numbers of women with tests are indicated in square brackets. ^a^ sum for twins; ^b^ tests consisted of simultaneous echocardiography and blood sampling for determination of biomarkers. *BMI* Body mass index, *BSA* Body surface area

**Table 2:** Clinical data normotensive pregnant women with singleton (*n* = 44) and twin (*n* = 22) pregnancies

	2nd trimester	3rd trimester	PP1	PP2
Singleton pregnancies (women tested)	(*n* = 42)	(*n* = 42)	(*n* = 41)	(*n* = 41)
**Twin pregnancies (women tested)**	**(*n*** **= 18)**	**(*n*** **= 21)**	**(*n*** **= 22)**	**(*n*** **= 22)**
**Clinical data**				
Maternal body weight, kg	SP 59.9 ± 8.1	63.2 ± 7.9	60.3 ± 8.5	57.7 ± 8.0
	**TP 56.8 ± 7.7**	**62.1 ± 8.7**	**56.8 ± 8.2**	52.2 ± 8.0 *
Weight gain, %	SP 10.1 ± 5.3	17.7 ± 7.5 **†**	12.1 ± 7.1	6.1 ± 6.1 **†**
	**TP 12.6 ± 6.3**	23.9 ± 9.4 *†	**13.6 ± 9.1**	**4.3 ± 0.9 †**
Heart rate, beats/min	SP 80 ± 10	83 ± 14	77 ± 11	68 ± 10 **†**
	**TP 77 ± 9**	**86 ± 14 †**	68 ± 10 *†	**68 ± 10 †**
Systolic blood pressure, mmHg	SP 107 ± 10	111 ± 11	109 ± 12	116 ± 13 **†**
	TP 98 ± 10 *	**108 ± 12 †**	**109 ± 12 †**	108 ± 11 *†
Diastolic blood pressure, mmHg	SP 62 ± 7	67 ± 9	64 ± 10	71 ± 11 **†**
	TP 58 ± 7 *	62 ± 9 *	**64 ± 9**	68 ± 7 *†

Data are presented as means ± SD; *, *P* < 0.05 vs. the value of singleton pregnancy. **†**, *P* < 0.05 vs. value of the same group determined in the 2nd trimester. *SP* Singleton pregnancies, *TP* Twin pregnancies

### Cardiac morphofunctional changes in women with singleton and twin pregnancies (Table 3, Fig. 2)

The left ventricular (LV) masses and left atrial (LA) volumes increased as gestation advanced, reaching their peak values at PP1. Regarding the ratio of early diastolic mitral flow velocity (E) to the average between early diastolic septal and lateral mitral annular velocities (e’) (E/e’) in women with singleton pregnancies, the value increased significantly at PP1 compared with that at the initial second trimester. However, the E/e’ values in women with twin pregnancies were higher than those in women with singleton pregnancies during the second trimester and at PP1 (*p* = 0.01 and *p* = 0.02, respectively). The LA volume corrected by body surface area was also significantly higher at PP1 in women with twin pregnancies than in women with singleton pregnancies (*p* = 0.04). Conversely, women with twin pregnancies had smaller average systolic septal and lateral mitral annular velocities (s’) as indexes of long-axis contractility than those with singleton pregnancies at PP1 and PP2 (*p* < 0.01 and *p* < 0.01, respectively), although the shortening fractions as an index of short-axis contractility were not significantly different between the study groups during the postpartum period (*p* = 0.52 and *p* = 0.66, respectively). During the postpartum period, women with twin pregnancies had transiently significantly lower heart rates and cardiac outputs than those with singleton pregnancies.

**Table 3:** Echocardiographic parameters in normotensive women with singleton (n = 44) and twin (n = 22) pregnancies

	2nd trimester	3rd trimester	PP1	PP2
Singleton pregnancies (*women tested*)	(*n* = 42)	(*n* = 42)	(*n* = 41)	(*n* = 41)
**Twin pregnancies (*women tested*)**	**(*n*** **= 18)**	**(*n*** **= 21)**	**(*n*** **= 22)**	**(*n*** **= 22)**
LVDd, mm	SP 46.0 (3.1)	46.2 (3.3)	47.0 (3.5)	45.4 (3.4)
	**TP 45.4 (2.5)**	**45.6 (4.1)**	**47.2 (2.9)**	**44.0 (3.1)**
LV ejection fraction (LVEF), %	SP 64.8 (4.3)	62.5 (4.1) **†**	63.6 (4.4)	61.8 (4.0) **†**
	**TP 65.7 (4.3)**	**63.1 (6.0)**	**65.3 (4.3)**	**62.6 (5.4)**
Shortening fraction, %	SP 35.5 (3.2)	33.6 (3.2) **†**	34.6 (3.6)	33.1 (3.2) **†**
	**TP 36.6 (3.4)**	**33.3 (4.6) †**	**35.3 (4.7)**	**33.4 (3.6)**
LV mass (LVM), g	SP 102.8 (19.0)	113.7 (23.6)	118.2 (26.1) **†**	102.7 (23.9)
	**TP 96.4 (12.9)**	**115.8 (15.6) †**	126.3 (19.4) *†	**94.5 (13.8)**
LVM/BSA (LVMI), g/m^2^	SP 63.6 (10.0)	68.8 (12.5)	73.1 (14.1) **†**	64.6 (13.0)
	**TP 61.8 (7.6)**	**71.3 (8.4) †**	80.9 (11.6) *†	**62.6 (7.4)**
LA diameter PLAX, mm	SP 31.9 (3.6)	32.3 (3.8)	33.6 (3.9) **†**	30.6 (3.8)
	**TP 31.4 (3.4)**	**33.7 (3.3)**	**34.3 (3.8) †**	**29.1 (3.8) †**
Maximum LA volume, mL	SP 38.6 (9.2)	40.2 (9.6)	45.9 (11.1) **†**	36.5 (9.8)
	**TP 40.3 (9.2)**	**45.7 (12.9)**	**51.0 (15.4) †**	**32.2 (10.8)**
Maximal LA volume/BSA (LAVI), mL/m^2^	SP 23.9 (5.2)	24.4 (5.7)	28.4 (6.1) **†**	22.9 (5.3)
	**TP 25.8 (5.4)**	28.0 (7.2) *	32.4 (8.9) *†	**21.2 (6.6)**
Inferior vena cava (IVC) diameter, mm	SP 13.6 (3.5)	10.9 (3.0) **†**	17.0 (4.0) **†**	15.3 (3.5)
	TP 11.2 (3.4) *	**9.3 (3.4)**	**15.8 (4.9) †**	13.0 (5.1) *
Stroke volume, mL	SP 68.2 (8.0)	65.8 (10.0)	74.5 (8.6) **†**	67.2 (8.5)
	**TP 67.0 (9.7)**	**75.7 (12.5)**	**74.0 (14.0)**	61.5 (8.4) *
Cardiac output (CO), L/min	SP 5.5 (0.8)	5.4 (0.9)	5.7 (0.9)	4.6 (0.7) **†**
	**TP 5.2 (0.9)**	**5.6 (1.1)**	4.9 (0.9) *	**4.3 (0.9) †**
CO/BSA (CI), L/min/m^2^	SP 3.4 (0.4)	3.3 (0.6)	3.5 (0.5)	2.9 (0.4) **†**
	**TP 3.3 (0.6)**	**3.5 (0.8)**	3.2 (0.6) *	**2.8 (0.5) †**
Systemic vascular resistance, dyn∙s/cm^5^	SP 1072 (143)	1157 (234)	1058 (195)	1441 (234) **†**
	**TP 1065 (233)**	**1066 (268)**	1222 (258) *	**1529 (325) †**
Mitral inflow parameters				
E, cm/s	SP 74 (15)	71 (18)	87 (16) **†**	69 (13)
	TP 84 (18) *	**79 (22)**	**92 (16)**	**67 (14) †**
A, cm/s	SP 53 (11)	56 (11)	58 (12)	52 (12)
	**TP 53 (10)**	**60 (9)**	**54 (14)**	**48 (11)**
E/A	SP 1.5 (0.4)	1.3 (0.4)	1.5 (0.4)	1.4 (0.4)
	**TP 1.6 (0.5)**	**1.3 (0.3) †**	**1.8 (0.6)**	**1.4 (0.3)**
Pulmonary vein inflow parameters				
S/D	SP 1.1 (0.3)	1.1 (0.3)	1.1 (0.3)	1.0 (0.3)
	**TP 1.0 (0.3)**	**1.1 (0.3)**	**1.0 (0.2)**	**0.9 (0.2)**
TDI mitral annulus				
Average				
s’ ave., cm/s	SP 11.3 (2.1)	11.5 (2.5)	10.6 (1.7)	10.1 (1.9) **†**
	**TP 10.6 (1.9)**	**10.6 (1.6)**	9.4 (1.2) *†	8.8 (1.0) *†
e’ ave. (e’), cm/s	SP 15.5 (2.2)	13.8 (2.1) **†**	14.5 (2.1)	13.6 (2.0) **†**
	**TP 14.8 (2.3)**	**13.3 (1.7)**	**13.7 (2.0)**	**14.2 (1.8)**
a’ ave., cm/s	SP 9.9 (1.8)	10.5 (2.0)	10.8 (2.3)	9.3 (2.0)
	**TP 10.3 (2.6)**	**11.0 (1.5)**	9.4 (2.2) *	**8.5 (1.8) †**
E/e’ average (E/e’)	SP 4.9 (1.1)	5.3 (1.6)	6.1 (1.2) **†**	5.1 (1.0)
	TP 5.9 (1.6) *	**6.0 (1.6)**	6.9 (1.5) *	**4.8 (0.9)**

Data are presented as means (SD); *, *P* < 0.05 vs. the value of singleton pregnancy. **†**, *P* < 0.05 vs. value of the same group determined in the 2nd trimester

*A* Late diastolic mitral flow velocity, *a’ ave*. Average of late diastolic septal and lateral mitral annular velocity, *BSA* Body surface area, *D* Anterograde diastolic pulmonary venous flow velocity, *E* Early diastolic mitral flow velocity, *e’ ave.* Average of early diastolic septal and lateral mitral annular velocity, *LA* Left atrium, *LV* Left ventricle, *LVDd* LV end-diastolic dimension, *PLAX* From parasternal long-axis view, *s’ ave*. Average of systolic septal and lateral mitral annular velocity, *S* Systolic pulmonary venous flow velocity, *SP* Singleton pregnancies, *TDI* Tissue Doppler imaging, *TP* Twin pregnancies

**Fig. 2:**
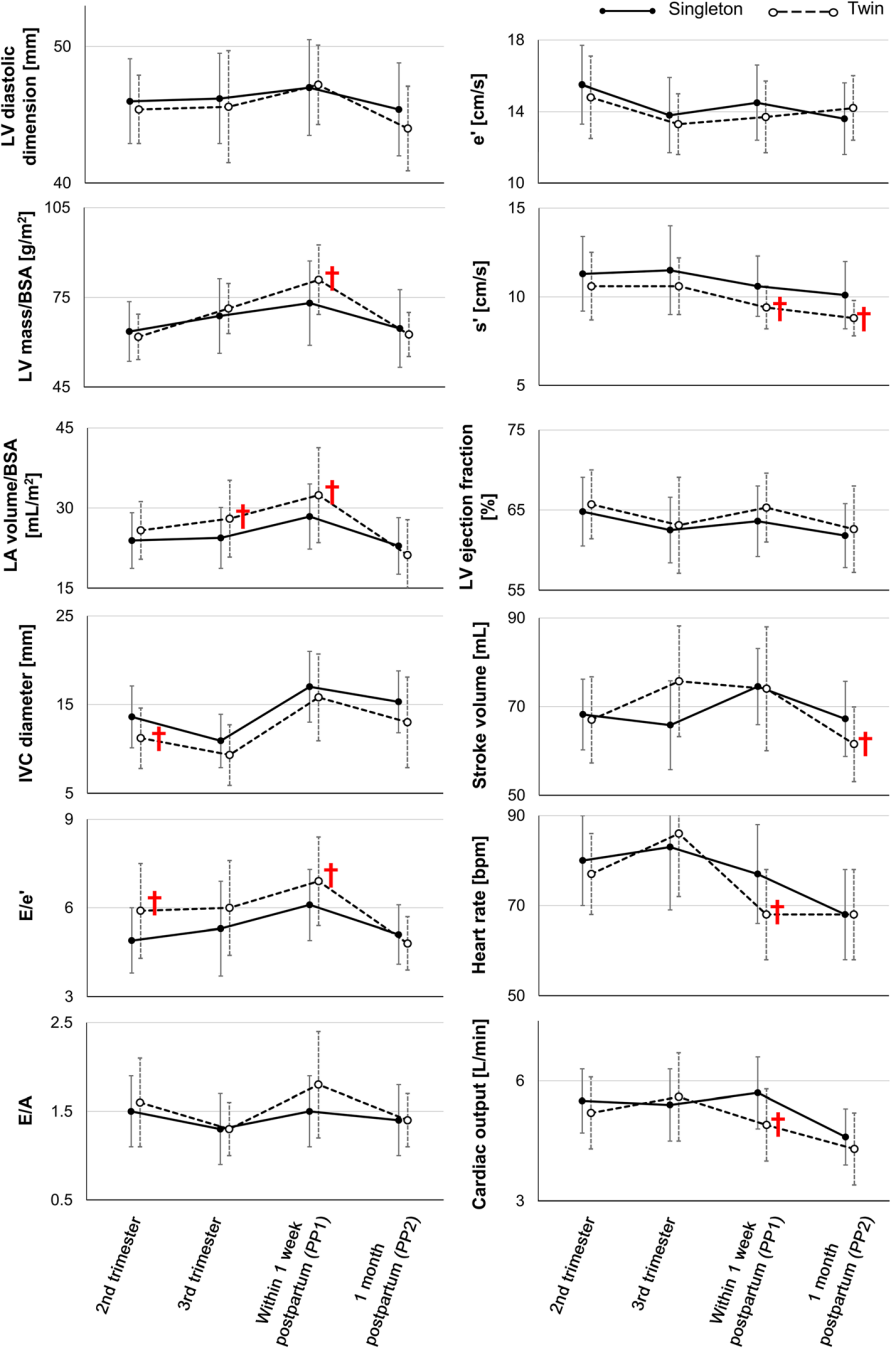
Cardiac morphofunctional changes in women with singleton and twin pregnancies. †, *p* < 0.05 between two groups. A, late diastolic mitral flow velocity; BSA, body surface area; E, early diastolic mitral flow velocity; e’, average of early diastolic septal and lateral mitral annular velocities; IVC, inferior vena cava; LA, left atrium; LV, left ventricle; s’, average of systolic septal and lateral mitral annular velocities. Table 3 lists the detailed data on these and other variables

### Changes in biomarkers in women with singleton and twin pregnancies (Table 4, Fig. 3)

The maternal weights were maximum during the third trimester, but the BNP and NT-proBNP levels reached their peak at PP1 and were significantly higher in women with twin pregnancies than in those with singleton pregnancies during the peripartum period. During this period, the hs-TnI level did not significantly change. Moreover, women with twin pregnancies had significantly higher serum creatinine levels and significantly lower eGFR than those with singleton pregnancies in the third trimester (*p* < 0.01 and *p* < 0.01, respectively).

**Table 4:** Blood parameters in normotensive women with singleton (n = 44) and twin (n = 22) pregnancies

	2nd trimester	3rd trimester	PP1	PP2
Singleton pregnancies (women tested)	(*n* = 42)	(*n* = 42)	(*n* = 41)	(*n* = 41)
**Twin pregnancies (women tested)**	**(*n*** **= 18)**	**(*n*** **= 21)**	**(*n*** **= 22)**	**(*n*** **= 22)**
BNP, pg/mL	SP 8.4 (5.2–11.3)	8.1 (2.9–13.3)	32.0 (15.1–52.6)* **†**	8.0 (3.6–13.4)
	**TP 11.0 (5.9–21.4)**	20.6 (10.0–29.6) *	56.9 (44.4–74.6) *†	**6.1 (2.9–12.2)**
NT-proBNP, pg/mL	SP 27 (19–52)	28 (13–49)	110 (44–218) **†**	30 (18–47)
	**TP 27 (18–48)**	49 (30–113) *	**180 (102–228) †**	**20 (14–48)**
hs-TnI, pg/mL	SP 0.9 (0.5–1.3)	1.2 (0.8–1.6)	1.9 (1.1–3.9) **†**	1.3 (1.0–1.6) **†**
	**TP 0.9 (0.2–1.8)**	**1.5 (1.0–2.2)**	**1.5 (1.3–4.7) †**	**0.9 (0.4–1.9)**
PRA, ng/mL/h	SP 20 (10–36)	15 (8.4–24)	3.6 (1.9–8.0) **†**	0.9 (0.5–1.8) **†**
	**TP 12 (5.6–20)**	**11 (5.1–19)**	**1.9 (0.9–5.9) †**	**1.3 (0.7–1.5) †**
PAC, pg/mL	SP 498 (368–747)	707 (441–1023)	96 (74.4–195) **†**	116 (77.8–161) **†**
	**TP 750 (439–1363)**	**503 (365–1435)**	75 (52–104) *†	**102 (58–142) †**
Serum creatinine, mg/dL	SP 0.47 (0.41–0.51)	0.49 (0.41–0.56)	0.55 (0.48–0.59) **†**	0.61 (0.54–0.66)v
	**TP 0.49 (0.40–0.53)**	0.54 (0.49–0.67) *†	**0.56 (0.49–0.63) †**	**0.62 (0.52–0.63) †**
eGFR, mL/min/1.73m^2^	SP 125 (109–138)	118 (99–140)	104 (92–120) **†**	91 (81–109) **†**
	**TP 120 (110–154)**	103 (86–118) *†	**101 (89–119) †**	**92 (89–110) †**

Data are presented as medians (25th – 75th); *, *P* < 0.05 vs. the value of singleton pregnancy. **†**, *P* < 0.05 vs. value of the same group determined in the 2nd trimester

*eGFR* Estimated glomerular filtration rate, *hs-TnI* High-sensitivity troponin I, *NT-proBNP* N-terminal fragment of precursor protein B-type natriuretic peptide, *PAC* Plasma aldosterone concentration, *PRA* Plasma renin activity, *SP* Singleton pregnancies, *TP* Twin pregnancies

**Fig. 3:**
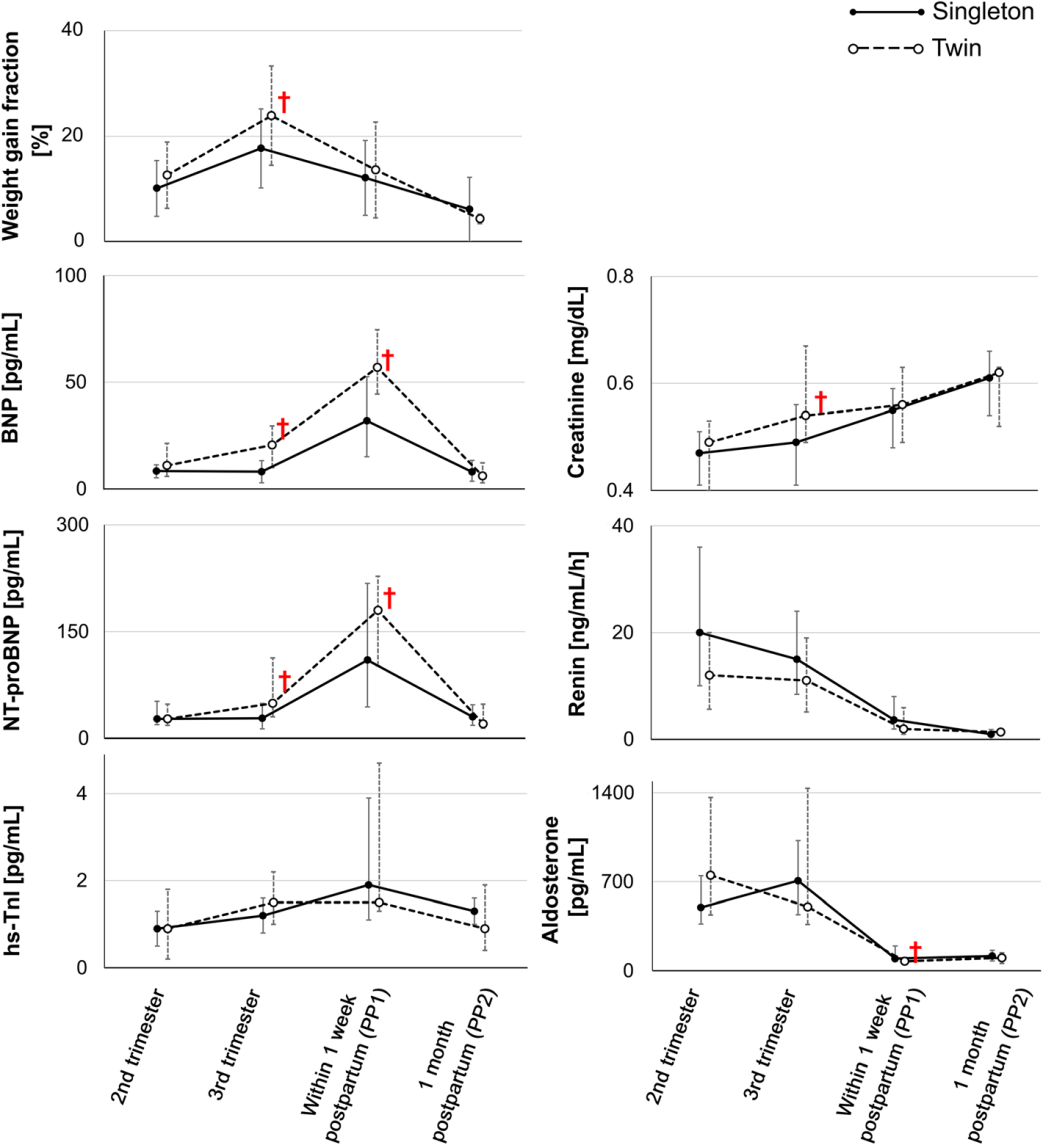
Changes in maternal body weight and biomarkers in women with singleton and twin pregnancies. †, *p* < 0.05 between two groups. BNP, brain natriuretic peptide; hs-TnI, high-sensitivity troponin I; NT-proBNP, N-terminal precursor protein BNP. See Tables 2 and 4 for detailed data on these and other variables

### Associations between cardiac biomarker levels and echocardiographic parameters (Fig. 4)

We analyzed correlations between standardized cardiac biomarkers, namely, BNP, NT-proBNP, and hs-TnI, and four echocardiographic parameters, namely, standardized s’, e’, E/e’, and LA volume index. BNP and NT-proBNP levels significantly positively correlated with E/e’ and LA volume index, whereas the hs-TnI level did not significantly correlate with any of the four echocardiographic parameters.

**Fig. 4:**
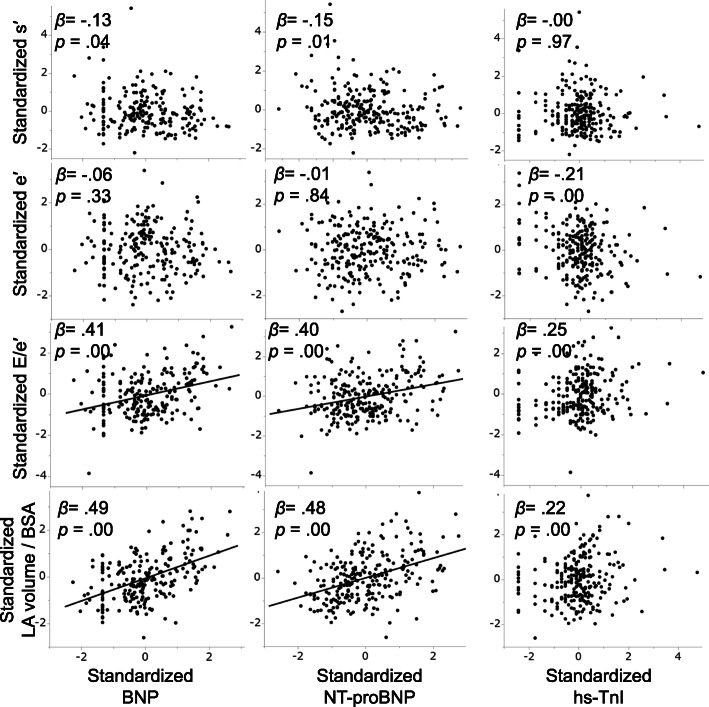
Standardized regression coefficients between blood variable levels and echocardiographic measurements. β, Standardized regression coefficient. Regression line drawn for β > 0.3. When the hs-TnI level was below the limit of detection (0.1 pg/mL), we assumed that hs-TnI was present at 0.1 pg/mL serum level. BSA, body surface area; BNP, brain natriuretic peptide; hs-TnI, high-sensitivity troponin I; NT-proBNP, N-terminal precursor protein BNP; E, early diastolic mitral flow velocity; e’, average of early diastolic septal and lateral mitral annular velocities; LA, left atrium; s’, average of systolic septal and lateral mitral annular velocities

### Correlations between BNP/NT-proBNP and hs-TnI during PP1 and later systolic and diastolic functions during PP2 (Fig. 5)

**Fig. 5:**
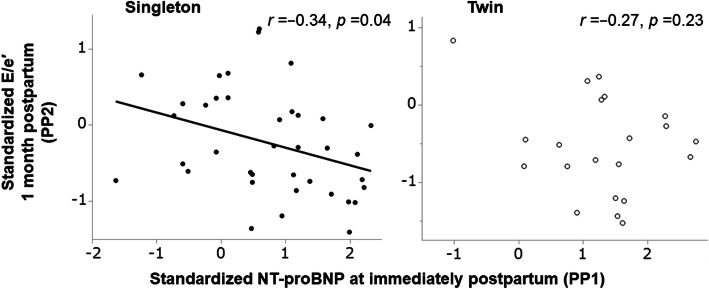
Correlations between NT-proBNP levels at immediate postpartum and later diastolic function at 1 month postpartum. NT-proBNP, N-terminal precursor protein BNP; E, early diastolic mitral flow velocities; e’, average of early diastolic septal and lateral mitral annular velocities

We analyzed standardized BNP, NT-proBNP, and hs-TnI levels at PP1 to detect their correlations with later standardized s’, e’, E/e’, and LA volume index values at PP2. The BNP/NT-proBNP levels at PP1 had a significantly negative correlation with later E/e’ values at PP2 in women with singleton pregnancies (r = − 0.34, *p* = 0.04), but no correlation was found in those with twin pregnancies (r = − 0.27, *p* = 0.23) (Fig. 5). However, the hs-TnI level did not significantly correlate with any of the four echocardiographic parameters.

### Differences in echocardiographic parameters and cardiac biomarkers according to chorionicity (Table 5)

We compared the echocardiographic parameters and cardiac biomarkers of monochorionic and dichorionic twins at each time point. In the second trimester, the monochorionic twins had a larger LA diameter (*p* = 0.03) and a smaller IVC diameter (*p* = 0.04) than diamniotic twins, but no significant difference was found in the LA volume (*p* = 0.88). Meanwhile, other echocardiographic parameters and cardiac biomarkers were not significantly different between the monochorionic and dichorionic twins.

**Table 5:** Echocardiographic parameters and cardiac biomarkers in monochorionic (n = 12) and dichorionic (n = 10) twin pregnancies

	2nd trimester	3rd trimester	PP1	PP2
monochorionic twins (*women tested*)	(*n* = 11)	(*n* = 11)	(*n* = 12)	(*n* = 12)
**dichorionic twins (*women tested*)**	**(*n*** **= 7)**	**(*n*** **= 10)**	**(*n*** **= 10)**	**(*n*** **= 10)**
LVDd, mm	mono 46 (2.0)	45 (4.2)	48 (2.6)	44 (3.0)
	**di 44 (2.7)**	**46 (4.2)**	**47 (3.4)**	**45 (3.3)**
LV ejection fraction (LVEF), %	mono 65 (5.0)	64 (7.3)	65 (4.6)	63 (5.6)
	**di 66 (3.0)**	**63 (4.5)**	**65 (4.1)**	**62 (5.4)**
LV mass (LVM), g	mono 101 (14)	120 (17)	131 (17)	97 (12)
	**di 89 (6.3)**	**111 (14)**	**120 (21)**	**91 (16)**
LVM/BSA (LVMI), g/m^2^	mono 64 (8.3)	73 (9.6)	83 (13)	64 (6.1)
	**di 58 (4.5)**	**70 (7.1)**	**78 (10)**	**61 (8.9)**
LA diameter PLAX, mm	mono 33 (3.3)	34 (2.6)	35 (2.9)	29 (3.2)
	di 29 (2.4) *	**34 (4.1)**	**33 (4.6)**	**29 (4.7)**
Maximum LA volume, mL	mono 40 (5.8)	45 (13)	54 (13)	34 (9.0)
	**di 41 (13.4)**	**46 (14)**	**47 (18)**	**31 (13)**
Maximal LA volume/BSA (LAVI), mL/m^2^	mono 26 (3.5)	27 (7.5)	34 (7.6)	22 (5.6)
	**di 26 (7.9)**	**29 (7.3)**	**30 (10)**	**20 (7.9)**
Inferior vena cava (IVC) diameter, mm	mono 10 (3.2)	8.8 (2.6)	15 (4.7)	13 (4.8)
	di 13 (2.7) *	**9.9 (4.2)**	**16 (5.4)**	**13 (5.8)**
Stroke volume, mL	mono 69 (9.7)	68 (15)	77 (17)	63 (9.7)
	**di 64 (9.7)**	**63 (9.2)**	**70 (8.9)**	**60 (6.9)**
Cardiac output (CO), L/min	mono 5.3 (0.9)	5.8 (1.3)	4.9 (0.9)	4.6 (0.9)
	**di 4.9 (0.9)**	**5.5 (0.9)**	**5.0 (1.0)**	**3.9 (0.9)**
CO/BSA (CI), L/min/m^2^	mono 3.4 (0.7)	3.6 (0.9)	3.1 (0.6)	3.0 (0.6)
	**di 3.2 (0.6)**	**3.4 (0.6)**	**3.2 (0.6)**	**2.6 (0.4)**
Systemic vascular resistance, dyn∙s/cm^5^	mono 1044 (261)	1023 (299)	1233 (271)	1502 (391)
	**di 1097 (197)**	**1108 (242)**	**1210 (258)**	**1559 (255)**
Mitral inflow parameters				
E/A	mono 1.7 (0.5)	1.3 (0.3)	1.9 (0.6)	1.3 (0.4)
	**di 1.6 (0.4)**	**1.3 (0.3)**	**1.8 (0.6)**	**1.5 (0.3)**
E/e’ average (E/e’)	mono 5.9 (1.9)	5.8 (1.6)	7.0 (1.4)	4.7 (0.8)
	**di 5.8 (1.1)**	**6.3 (1.7)**	**6.7 (1.5)**	**4.8 (1.0)**
**Cardiac biomarkers**				
BNP, pg/mL	mono 11 (5.1–22)	15 (6.4–24)	62 (54–89)	5.0 (1.9–9.5)
	**di 11.4 (5.8–21)**	**21 (10–41)**	**45 (21–66)**	**9.2 (4.7–17)**
NT-proBNP, pg/mL	mono 27 (18–48)	34 (24–107)	203 (129–381)	17 (9.5–24)
	**di 27 (25–93)**	**53 (42–134)**	**160 (72–219)**	**38 (19–80)**
hs-TnI, pg/mL	mono 1.2 (0.4–2.0)	1.5 (1.0–2.1)	2.1 (1.4–11)	1.7 (0.7–2.0)
	**di 0.5 (0.1–1.3)**	**1.5 (1.0–2.4)**	**1.4 (0.9–2.1)**	**0.4 (0.2–1.2)**

Data are presented as means (SD) for echocardiographic parameters and as medians (25th – 75th) for cardiac biomarkers; *, *P* < 0.05 vs. the value of monochorionic twin pregnancy

*A* Late diastolic mitral flow velocity, *BNP* B-type natriuretic peptide, *BSA* Body surface area, *di* Dichorionic twins, *E* Early diastolic mitral flow velocity, *e’ ave*. Average of early diastolic septal and lateral mitral annular velocity, *hs-TnI* High-sensitivity troponin I, *LA* Left atrium, *LV* Left ventricle, *LVDd* LV end-diastolic dimension, *mono* Monochorionic twins, *NT-proBNP* N-terminal fragment of precursor protein B-type natriuretic peptide, *PLAX* From parasternal long-axis view

## Discussion

Our study obtained the following findings: (1) women with singleton and twin pregnancies reached the peak levels of LA volume index, E/e’, and BNP/NT-proBNP at PP1; (2) women with twin pregnancies had higher LA volume index, E/e’, and BNP/NT-proBNP level and smaller s’ than those with singleton pregnancies at PP1; (3) women with twin pregnancies had significantly higher serum creatinine level and significantly lower eGFR than those with singleton pregnancies in the third trimester; (4) the BNP/NT-proBNP level had a significantly positive correlation with the LA volume index and E/e’, (5) the BNP/NT-proBNP level at PP1 predicted diastolic function recovery at PP2 in women with singleton pregnancy.

The LA volume index, E/e’, and BNP/NT-proBNP variables reached their peak at PP1. Therefore, regardless of the number of fetuses, the maximal cardiac volume load occurred at PP1 and not during the late stages of pregnancy. This concept is consistent with our previous reports [11, 24], which suggested that the maximum cardiac volume load occurred at PP1 in normotensive as well as hypertensive women with singleton pregnancies. These results are also consistent with Burlingame et al.’s study [12] and explain the likelihood of heart failure at PP1 among women with structural heart diseases [27]; peripartum cardiomyopathy is also often observed during the postpartum period [5]. Interstitial fluid retention occurs physiologically even in normotensive singleton pregnancies, wherein approximately 40% women exhibit pitting edema during the third trimester [28]. The decrease in systemic vascular resistance [10] causes an increase in reserved blood at the splanchnic venous reservoir [29]. Parturition is thought to reverse this process. In contrast, the increase in systemic vascular resistance during the postpartum period [10] can actively expel splanchnic blood into the systemic circulation [29], resulting in a maximal volume load in the early postpartum period.

In this study, the LA volume index, E/e’, and BNP/NT-proBNP level were higher in women with twin pregnancies than in those with singleton pregnancies at PP1. Thus, twin pregnancy is a prominent risk factor for peripartum cardiomyopathy [5]. In Ghi et al.’s study, LV diastolic function recovered 6 months after childbirth, but LV contractility decreased [17]. Consistent with their findings, our results showed that e’ was similar to that in singleton pregnancy but s’, which is the contractility in the longitudinal direction, was lower than that in singleton pregnancy until PP2 [17]. Moreover, Orabona et al. reported a reduction in preload reserve in twin pregnancy [18]. Considering that the circulating blood volume was higher in twin pregnancies than in singleton pregnancies [1], this result was caused by the fact that the volume load during pregnancy exceeded the physiological limit of reversible adaptation.

To our knowledge, this study is the first to report that women with twin pregnancies had significantly higher serum creatinine levels and lower eGFRs than those with singleton pregnancies during third trimester (Table 4 and Fig. 3). The lower eGFR in women with twin pregnancies involves certain mechanisms. For instance, the central venous pressure may be higher in women with twin pregnancies than in those with singleton pregnancies owing to the smaller IVC diameter (compressed by the enlarged uterus) in women with twin pregnancies (Fig. 2) despite higher blood volume expansion [1]. Despite the higher blood volume expansion in women with twin pregnancies than in those with singleton pregnancies [1], the third-trimester cardiac outputs were similar between these two groups with similar heart rates (Fig. 2); although in some studies, women with twin pregnancies had a higher cardiac output than those with singleton pregnancies [13–16]. Therefore, blood flow velocities were slower in twin pregnancies than in singleton pregnancy, resulting in lower eGFRs in the former than in the latter.

Moreover, the BNP/NT-proBNP level significantly positively correlated with the LA volume index and E/e’ (Fig. 4), implying that BNP is secreted by tension applied to the myocardium with volume and pressure overload [30]. Nonhemodynamic factors such as catecholamines and other neuroendocrines synthesize BNP [31]. In pregnant women, the mechanism of BNP secretion is insufficiently understood, but BNP may be secreted by diuretic action to reduce the volume and pressure overload on the myocardial wall [32, 33]. In the present study, in singleton pregnancies, the higher the BNP level immediately after childbirth was, the lower the E/e’ at PP2 (Fig. 5). Therefore, BNP has a cardioprotective effect in healthy pregnant women with singleton pregnancies. In addition, a higher BNP level immediately postpartum predicted diastolic function recovery at PP2, as evidenced by E/e’ decrease as an index of the LV filling pressure (Fig. 5). This phenomenon may also mean that BNP is actively secreted to protect cardiac function from the cardiac volume load immediately postpartum. However, this trend was not observed in women with twin pregnancies because of the limited sample size and because catecholamine production from postoperative pain affected BNP, considering that 95% of women had undergone cesarean section [34].

We found two reports of hemodynamic comparison between monochorionic and dichorionic twin pregnancies [18, 35]. Ghi et al. reported that monochorionic twin pregnancies had lower cardiac output, higher systemic vascular resistance, and higher E/A than dichorionic twin pregnancies [35], but our study found no differences in echocardiographic parameters and cardiac biomarkers between monochorionic and dichorionic twin pregnancies (Table 5), as in the results of Orland et al. [18]. Ghi et al. [35] attributed the difference to the lower circulating blood volume of the monochorionic twin pregnancies, compared with that of the dichorionic twin pregnancies, but our study had no data to complement their findings. This result may also be associated with sample size issues; hence, further investigation is required.

This study has few limitations. First, we missed the first-trimester twin pregnancy data. As a regional core center, our hospital often accepts referred women with twin pregnancies after the first trimester. Second, twin pregnancies had a higher cesarean section rate than singleton pregnancies. Given that cesarean delivery results in a greater intravascular burden after childbirth than vaginal delivery [11], cesarean section may influence the postpartum course of twin pregnancies. Third, our study had a limited study population and was conducted at a single center, potentially limiting the generalizability of our findings.

## Conclusions

In conclusion, hemodynamic changes were considerably different between women with singleton pregnancies and twin pregnancies. Women with twin pregnancies had a greater cardiac load at peripartum; hence, twin pregnancy is a consistent and prominent risk factor for heart failure. Although the mechanism of BNP secretion in pregnant women remains unclear, the BNP/NT-proBNP level had a significantly positive correlation with the LA volume index and E/e’, and BNP secretion may reduce the volume overload and pressure overload on the myocardial wall by diuretic action.

## Data Availability

The datasets cannot be made publicly available due to ethical restrictions by the Institutional Review Board of Hokkaido University Hospital. Please contact the corresponding author to consider the data set disclosure.

## Abbreviations

BNP: Brain natriuretic peptide
E: Early diastolic mitral flow velocity
e’: Average between early diastolic septal and lateral mitral annular velocities
eGFR: Estimated glomerular infiltration rate
hs-TnI: High-sensitivity troponin I
IVC: Inferior vena cava
LA: Left atrium
LV: Left ventricle
NT-proBNP: N-terminal precursor protein BNP
PP1: Within 1 week after childbirth
PP2: 1 month after childbirth
